# Dietary micro-fibrillated cellulose improves growth, reduces diarrhea, modulates gut microbiota, and increases butyrate production in post-weaning piglets

**DOI:** 10.1038/s41598-023-33291-z

**Published:** 2023-04-16

**Authors:** Md Karim Uddin, Md. Rayhan Mahmud, Shah Hasan, Olli Peltoniemi, Claudio Oliviero

**Affiliations:** Department of Production Animal Medicine, Faculty of Veterinary Medicine, University of Helsinki, Helsinki, Finland

**Keywords:** Microbiome, Animal physiology, Phylogeny, Statistical methods, Microbiota, Diarrhoea

## Abstract

Dietary fiber (DF) supplementation is one of the strategies to prevent on-farm infections; it has the capability to improve gut health and piglet performance. Among the beneficial DFs, micro-fibrillated cellulose (MFC) is a new-generation plant-derived innovative feed ingredient; MFC, originating from sugar-beet pulp, has a hyper-branched structure with the ability to form shear-thinning hydrogel and has a high water-binding capacity. We aimed to determine the effects of MFC supplementation on piglets' performance before and after weaning. We included 45 sows and their piglets in this trial and monitored the results until the piglets were 7 weeks old. Piglets supplemented with MFC had higher body weight and average daily growth (ADG) than did control piglets, both pre- and post-weaning. In addition, MFC supplementation in post-weaning piglets improved butyrate content, and reduced diarrhea incidence. These phenomena, perhaps due to the MFC supplementation at different stages until age 7 weeks. In addition, after weaning, MFC supplementation stimulated the growth of butyrate-producing bacteria such as *Ruminococcus.2, Ruminococcaceae.UCG.014, Intestinibacter*, *Roseburia,* and *Oribacterium* genera, as well as reduced the pathogenic bacteria, such as *Campylobacter*, and *Escherichia*. Evidently, supplementation of MFC in feed to young piglets can improve growth performance and butyric acid content and reduce post-weaning diarrhea.

## Introduction

Piglets undergo stress during weaning that is called weaning stress. This results from the immaturity of their digestive and immune systems, affected by changes in their environment and feed, and this then results in low feed intake, minimal weight gain, and diarrhea^1^. In modern pig production, various feeding strategies now popular reduce diarrhea incidence at weaning, and improve production performance. Such strategies include administration of prebiotics, probiotics, fatty acids, organic acids, essential oils, and dietary fibers^2,3^.

Like other feeding strategies, supplementation with dietary fiber (DF) is one of the regimes implemented at various stages of pig production^2^. Dietary fiber plays a crucial role in maintaining diversified gut microbiota and thus human and animal gut health^4^. Adding a high-fiber diet can surge the activity of fibrolytic bacteria in the large intestine of growing pigs^5^, and a high volume of cellulolytic bacteria favors the establishment and development of some beneficial bacteria, meanwhile reducing harmful bacteria, which is advantageous to gut health and seemingly exerts a prebiotic effect^6^.

In addition, cellulolytic bacteria produce short-chain fatty acids (SCFAs), principally acetate, propionate, and butyrate^7^. These SCFA produced in the large intestine are estimated to contribute 5% to 15% of the total human caloric requirements^8^, while in pigs providing approximately 24% of the energy for their body’s thermoregulation^9^. Approximately 15% of the maintenance energy requirement of growing pigs and 30% in gestating sows comes from large-intestine SCFA^10^. Moreover, SCFAs originating from DF fermentation, especially butyrate, demonstrate numerous health benefits, including acting as the main energy source for colonocytes, influencing immune system regulation, and reducing inflammation^11^. Moreover, DF leads to increased abundance of Lactobacilli and reduces coliform abundance in the small intestine^12^.

Furthermore, inclusion of moderate amounts of insoluble fiber sources in the diets of young pigs with compromised hygienic and health status, may reduce the incidence of their post-weaning diarrhea (PWD) in the first 2 weeks^13^. DF supplementation can alleviate piglet weaning stress by improving bacterial diversity and rapidly stabilizing the gut microbial community^14^, improving piglet gut environment in the delicate phase of weaning. Sugar beet pulp (SBP), a pectin-rich fiber, contains nitrogen-free leachate, crude protein (CP), and high-quality crude fiber (CF) including a high quantity of l-arabinose polymer^15^. Due to its highly soluble fiber content, SBP is easily digested in the porcine gut^16^.

Micro-fibrillated cellulose (MFC), a new-generation plant-derived innovative feed ingredient, originated from sugar-beet pulp with a hyper-branched structure consisting of more than 90% dry matter^17^. It has the ability to form shear-thinning hydrogel with a high water-binding capacity^18^. In addition, cellulose hydrogel is widely applied for tissue regeneration of bone, cartilage, and neural tissues, because of its biocompatibility^19^.

The gut microbiota play a crucial role in piglet health and nutrition^20^. Their microbial composition can undergo modulation by various factors such as the maternal microbiota^21^, piglet age and health status, environmental factors, growth promoters^22^, and the feeding regimes^23^. Among such regimes, fiber supplementation plays a significant role in gut microbiota development and in improved intestinal integrity^24^. Researchers exploring the relationship between dietary fiber and pig gut microbiota found fiber supplementation causing changes in feed efficiency^25^. Moreover, a diet lacking in fiber is associated with impaired intestinal-barrier function of the colonic mucosa and with higher pathogen susceptibility^26^. Fibers such as SBP supplemented to piglets lead to increased large intestine weight^27^ and reduction in fecal *Enterococcus* spp^28^. In addition, they lead to increased abundance of *Lactobacillus* and inhibit the colonization of coliform bacteria^29^, which, in weaned piglets, leads to reduced incidence of post-weaning diarrhea^30^. However, many studies investigated the direct effects of fiber diets on the piglet performance. Research reports on the indirect maternal effects (when sows received fiber during late pregnancy) and the direct dietary effects (when piglets received fiber through the creep feed and post weaning feed) of innovative feed ingredients, MFC, on piglets’ performance are scarce.

We hypothesized that MFC supplementation to sows and piglets improves piglet performance at birth and during weaning and post-weaning, in regard to body weight, microbiota modulation, intestinal SCFA increase, and to reduction in diarrhea incidence and in piglet mortality.

## Results

### Effects of MFC on piglet body weight

**Figure 1:**
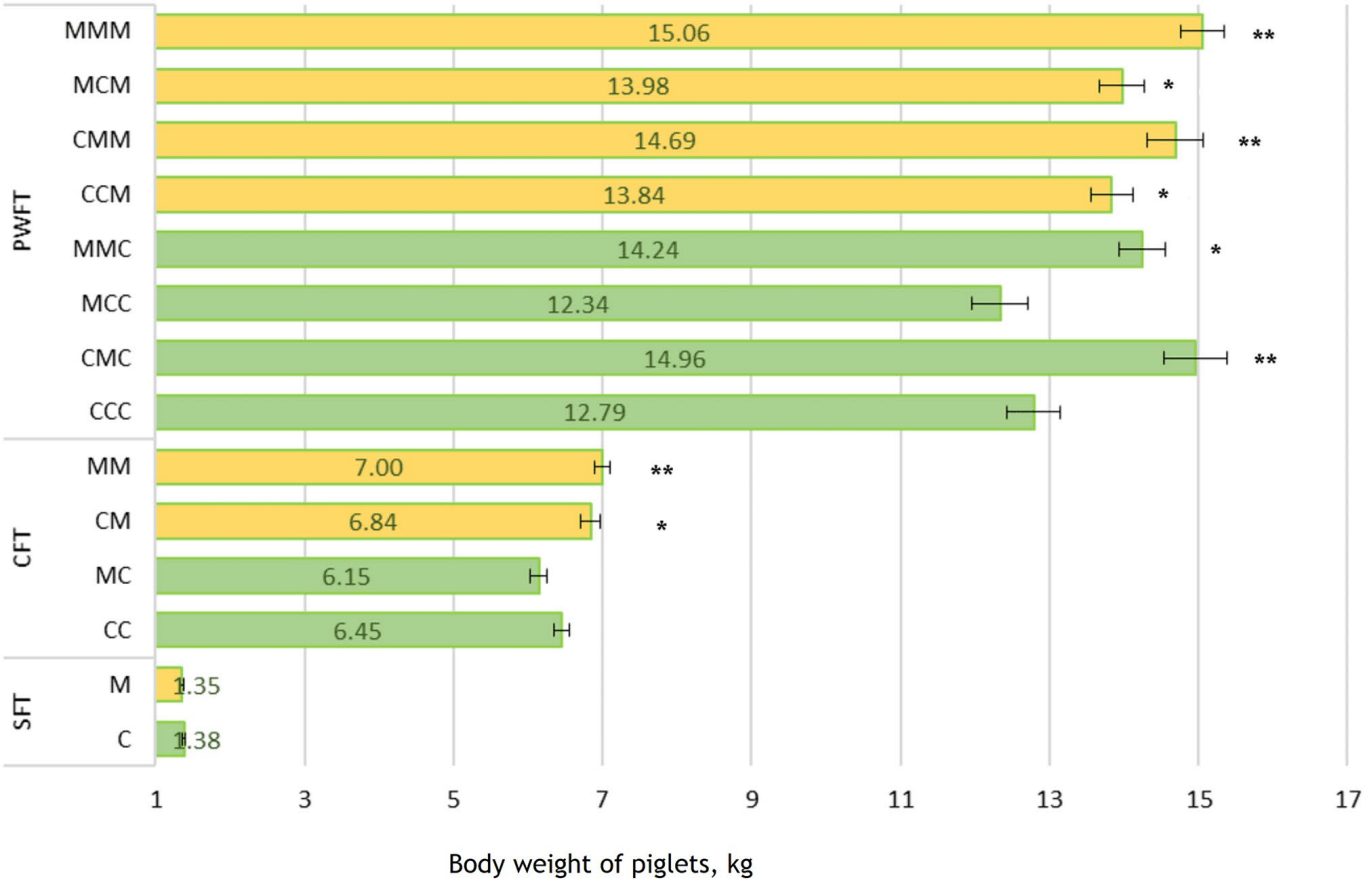
Body weights of piglets at different ages. *MFC* Sows fed micro-fibrillated cellulose; *MM* sow MFC, piglets fed MFC creep feed; *MC* sow MFC, piglets fed control creep feed; *CM* sow control, piglets fed MFC creep feeding; *CC* sow control, piglets fed control creep feed. *C* Control feed of sows; *M* MFC feed of sows; *SFT* Sow feeding treatment; *CFT* Creep feed treatment piglets; *PWFT* Post-weaning feed treatment piglets. *MMM* sow MFC, piglets fed MFC creep feed, post-weaning piglets MFC feeding; *MMC* sow MFC, piglets fed MFC creep feed, post-weaning piglets control feed; *MCM* sow MFC, piglets fed control creep feed, post-weaning MFC feed; *MCC* sow MFC, piglets fed control creep feed, post-weaning control feed; *CMM * sow control feed, piglets fed MFC creep feed, piglets post-weaning MFC feed; *CMC* sow control feed, piglets fed MFC creep feed; piglets post-weaning control feed; *CCM* sow control feed, piglets fed control creep feeding, piglets post-weaning MFC feed; *CCC* sow control feed, piglets fed control creep feed, piglets post-weaning control feed. *P < 0.05, and **P < 0.01.

At weaning, at 3 weeks of age, body weight for piglet groups CM and MM was higher than for CC groups, and the MC group showed a lower body weight than that of CC piglets (Fig. 1). Post-weaning, at 7 weeks of age, the body weights of MMM, MCM, CMM, CCM, MMC, and CMC piglets were higher than the weight of CCC piglets, whereas no significant difference emerged between CCC and MCC piglets (Fig. 1).

### Effects of MFC on piglet ADG

In the case of ADG, CM and MM showed higher body growth than did CC groups, and the MC group showed lower body growth than did CC piglets during weaning at 3 weeks of age (Table 1). Post-weaning, at 7 weeks, MMM, MCM, CMM, CCM, MMC, and CMC piglets had higher ADG than did CCC piglets, whereas no significant difference in ADG appeared between CCC and MCC piglets (Table 1).

**Table 1:** Piglet growth at weaning and post-weaning.

Treatment	Treatment	ADG	P-value^b^	P-value^a^
Creep feed treatment	CC	307 ± 0.11		< 0.01
	MC	293 ± 0.11	0.06	
	CM	326 ± 0.13	0.02	
	MM	333 ± 0.11	< 0.01	
Post-weaning feed treatment	CCC	261 ± 0.36		< 0.01
	CMC	305 ± 0.42	< 0.01	
	MCC	252 ± 0.38	0.35	
	MMC	291 ± 0.31	< 0.01	
	CCM	282 ± 0.29	0.03	
	CMM	300 ± 0.38	< 0.01	
	MCM	285 ± 0.30	0.01	
	MMM	307 ± 0.29	< 0.01	

*ADG* average daily growth; *MM* sow MFC, piglets fed MFC creep feed; *MC* sow MFC, piglets fed control creep feed; *CM* sow control, piglets fed MFC creep feed; *CC* sow control, piglets fed control creep feed. *MMM* sow MFC, piglets fed MFC creep feed, post-weaning piglets MFC feed; *MMC* sow MFC, piglets fed MFC creep feed, post-weaning piglets control feed; *MCM* sow MFC, piglets fed control creep feed, post-weaning MFC feed; *MCC* sow MFC, piglets fed control creep feed, post-weaning control feed; *CMM* sow control feed, piglets fed MFC creep feed, piglets post-weaning MFC feed; *CMC* sow control feed, piglets fed MFC creep feed, piglets post-weaning control feed; *CCM* sow control feed, piglets fed control creep feed, piglets post-weaning MFC feed; *CCC* sow control feed, piglets fed control creep feed, piglets post-weaning control feed.

^a^Overall p-value.

^b^Group p-value.

### Volatile fatty acids

At weaning, no significant differences were observable in total VFA, butyric acid, isobutyric acid, 2-mebutyric acid, 3-mebutyric acid, pentanoic acid, hexanoic acid, and propionic acid between control (CC), and MFC (MM) groups (Table 2). At post-weaning, at age 7 weeks, hexanoic acids level tended to be higher in MFC-treated piglets (MMM) than in control piglets (CCC), but butyric acid was higher in MFC-treated piglets than in control piglets (Table 2). No significant differences were evident for total VFA, acetic acid, propionic acid, isobutyric acid, 2-mebutyric acid, 3-mebutyric acid, and pentanoic acid (Table 2).

**Table 2:** Effects of MFC supplementation on fecal volatile fatty acids content of pre-weaning and post-weaning piglets.

VFA, mg/kg	Preweaning			Post-weaning		
	Control (55)	MFC (64)	P-value	Control (29)	MFC (34)	P-value
Total VFA	4128.9 ± 300.96	4344.15 ± 246.31	0.58	6022.18 ± 255.55	6552.09 ± 244.8	0.14
Acetic acid	1600.61 ± 86.33	1700.94 ± 81.71	0.40	3036.9 ± 116.14	3097.91 ± 96.35	0.68
Propionic acid	933.93 ± 59.97	1066.04 ± 69.76	0.16	1537.35 ± 75.87	1661.2 ± 89.23	0.30
Butyric acid	650.37 ± 92.15	592.76 ± 58.89	0.59	984.19 ± 62.95	1287.13 ± 76.63	** < 0.01**
IsoButyric acid	202.5 ± 17.8	204.19 ± 14.08	0.94	81.76 ± 9.28	92.16 ± 6.72	0.35
2-MeButyric acid	170.29 ± 15.19	171.72 ± 12.32	0.94	50.07 ± 7.28	57.63 ± 5.53	0.40
3-MeButyric acid	240.45 ± 21.49	246.75 ± 18.40	0.82	70.25 ± 9.27	78.71 ± 6.37	0.44
Pentanoic acid	284.05 ± 26.08	311.56 ± 21.17	0.41	211.29 ± 14.82	212.92 ± 9.9	0.92
Hexanoic acid	46.69 ± 3.48	50.19 ± 4.08	0.52	50.36 ± 5.55	64.42 ± 4.76	0.06

*VFA* volatile fatty acid; *Control* CC at weaning, and CCC at post-weaning; *MFC* MM at weaning, and MMM at post-weaning.

### Diarrhea incidence

**Figure 2:**
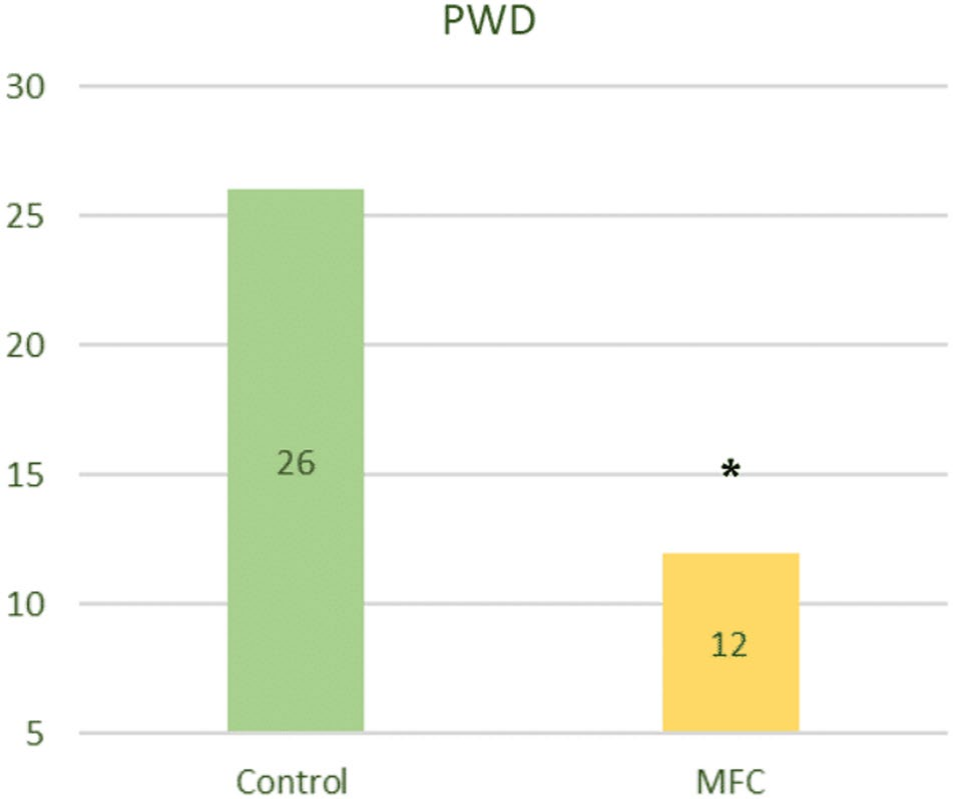
Piglet diarrhea cases at 7 weeks of age (PWD). Asterisks indicate the statistically significant differences (p < 0.05).

We observed no significant differences in diarrhea incidence before weaning among the 4 treatment groups that received control (CC and MC) or MFC (CM and MM) diets (P > 0.05). Interestingly, at 7 weeks, diarrhea incidence tended to be lower in the CCM, CMM, and MMM groups (P < 0.1), whereas the MCM group had lower diarrhea incidence than the CCC group. The remaining MMC, MCC, and CMC groups showed no significant differences from the CCC group (Fig. 2).

### Piglet mortality

We observed no differences in piglet mortality at age 3 weeks. At 7 weeks, no significant difference emerged between MFC (MMM, MCM, CMM, and CCM) and the control diet (MMC, MCC, CMC, and CCC).

## Microbiome results

### Microbiome diversity

**Figure 3:**
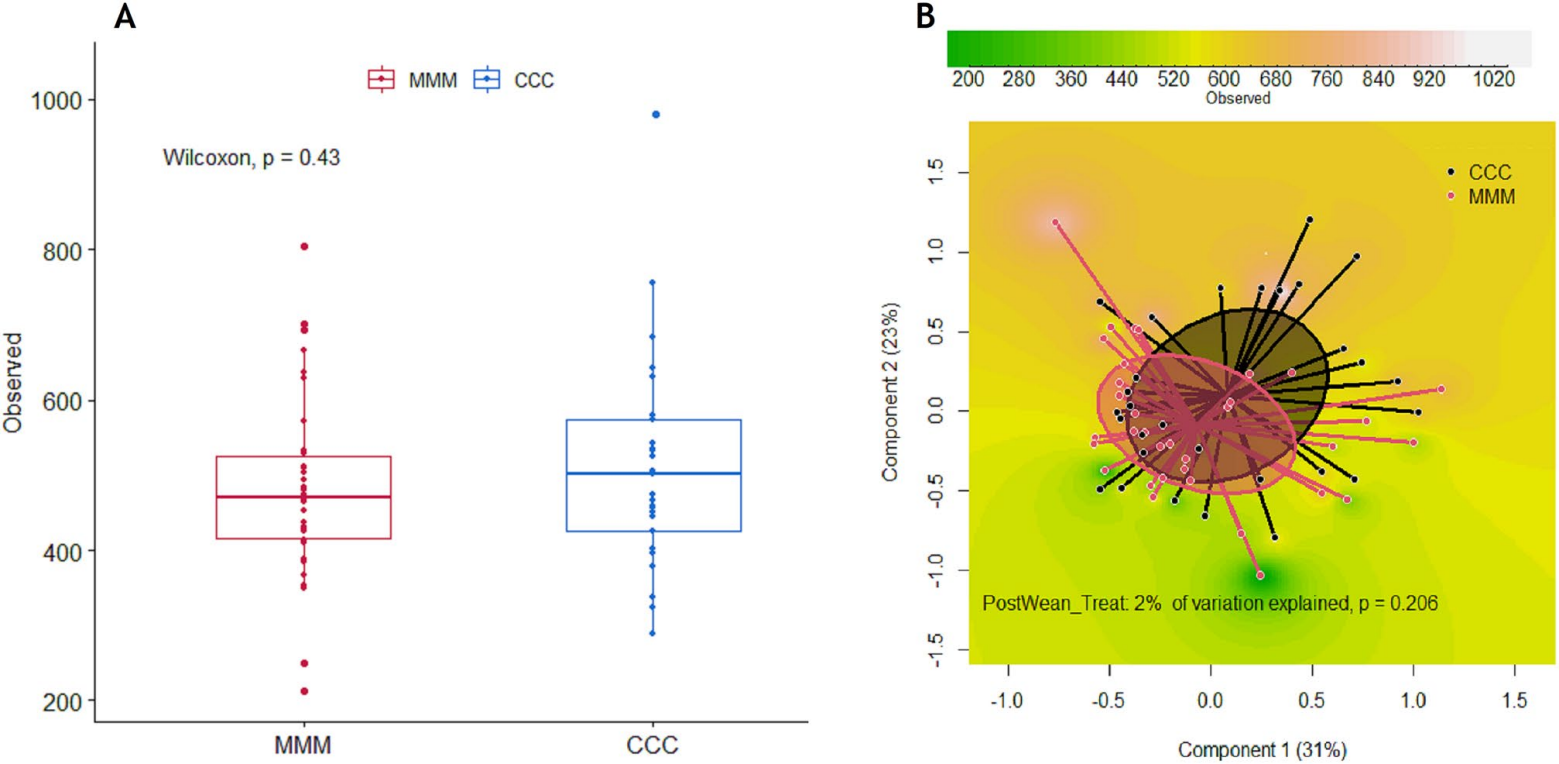
Diversity of gut microbiota of control and MFC piglets. (**A**) Alpha diversity (observed index), and (**B**) Principal coordinates analysis (PCoA) plot.

We estimated alpha diversity and observed no differences between groups (Fig. 3). We measured the beta-diversity (PcoA) with bray curtis distance and found no differences between control and MFC groups (Fig. 3).

### Effects of MFC on fecal microbiota

**Figure 4:**
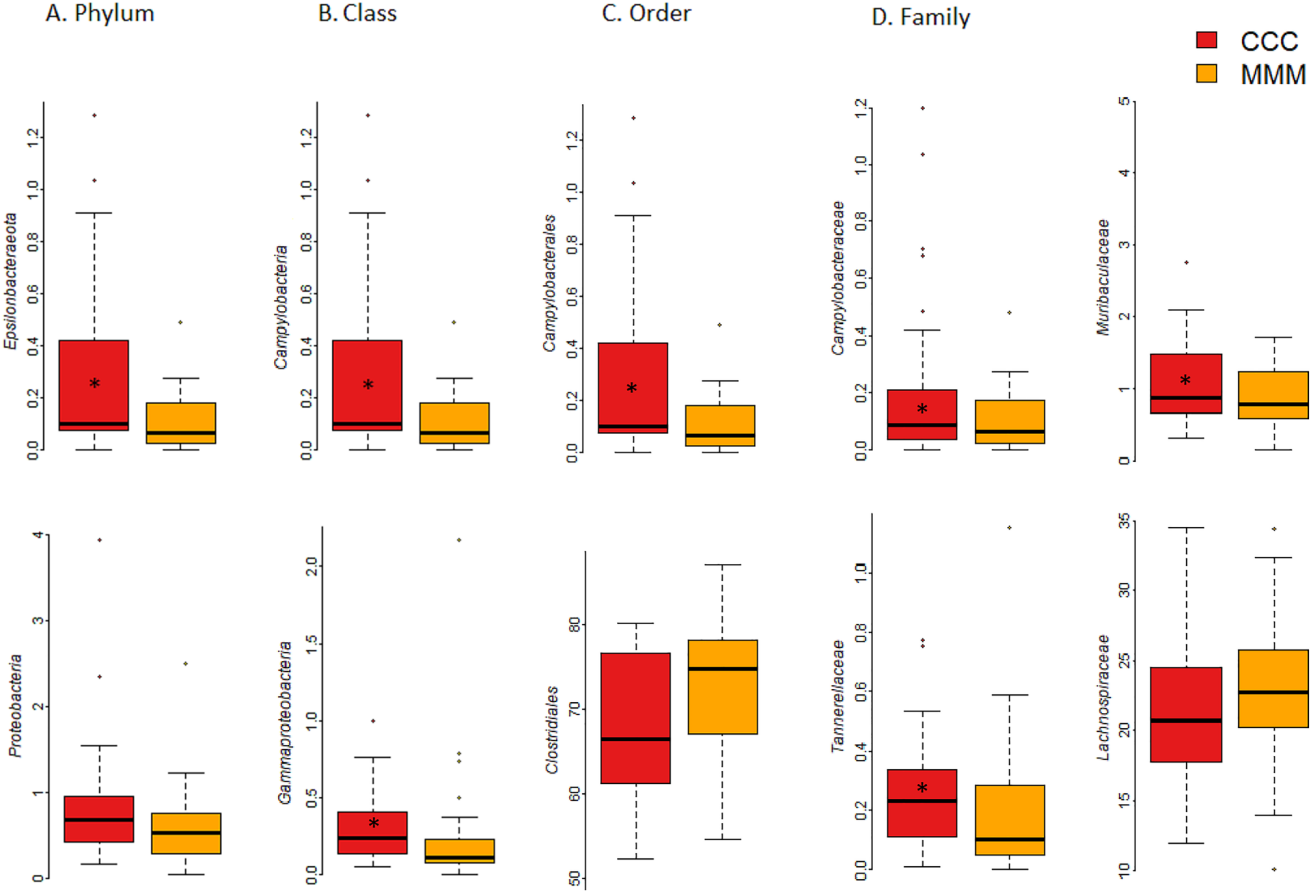
Relative abundances of microbiota between control (CCC), and MFC (MMM) piglet groups at different taxa. (**A**) Phylum, (**B**) Class, (**C**) Order, (**D**) Family. Asterisks indicate significant differences (p < 0.05).

For the microbiota results interpretation, we considered the findings significant with P values lower than 0.05, tending to significant with P values between 0.05 and 0.10. At phylum level, relative abundances of Epsilonbacteraeota were significantly higher in the control group (CCC) than in the MFC group (MMM), whereas Proteobacteria tended to be higher in the control group (Fig. 4, Supplementary Table S1). At class level, higher abundances of Campylobacteria and Gammaproteobacteria classes occurred among the control piglets.

In addition, Clostridia tended to be higher in the MFC (p < 0.1) and Bacilli higher in the control group (Fig. 4). Campylobacteriales was significantly higher in the control group (Fig. 4). Clostridiales tended to be higher in MFC, and Lactobacillales in the control group (Supplementary Table S1). As for the control group at family level, Campylobacteraceae, Tannerellaceae, Family_XIII, and Muribaculaceae were higher, and the Streptococcaceae and Rikenellaceae families tended to be higher than in the MFC group; the Lachnospiraceae family abundances tended, however, to be higher in the MFC group (Fig. 4, Supplementary Table S1).

**Figure 5:**
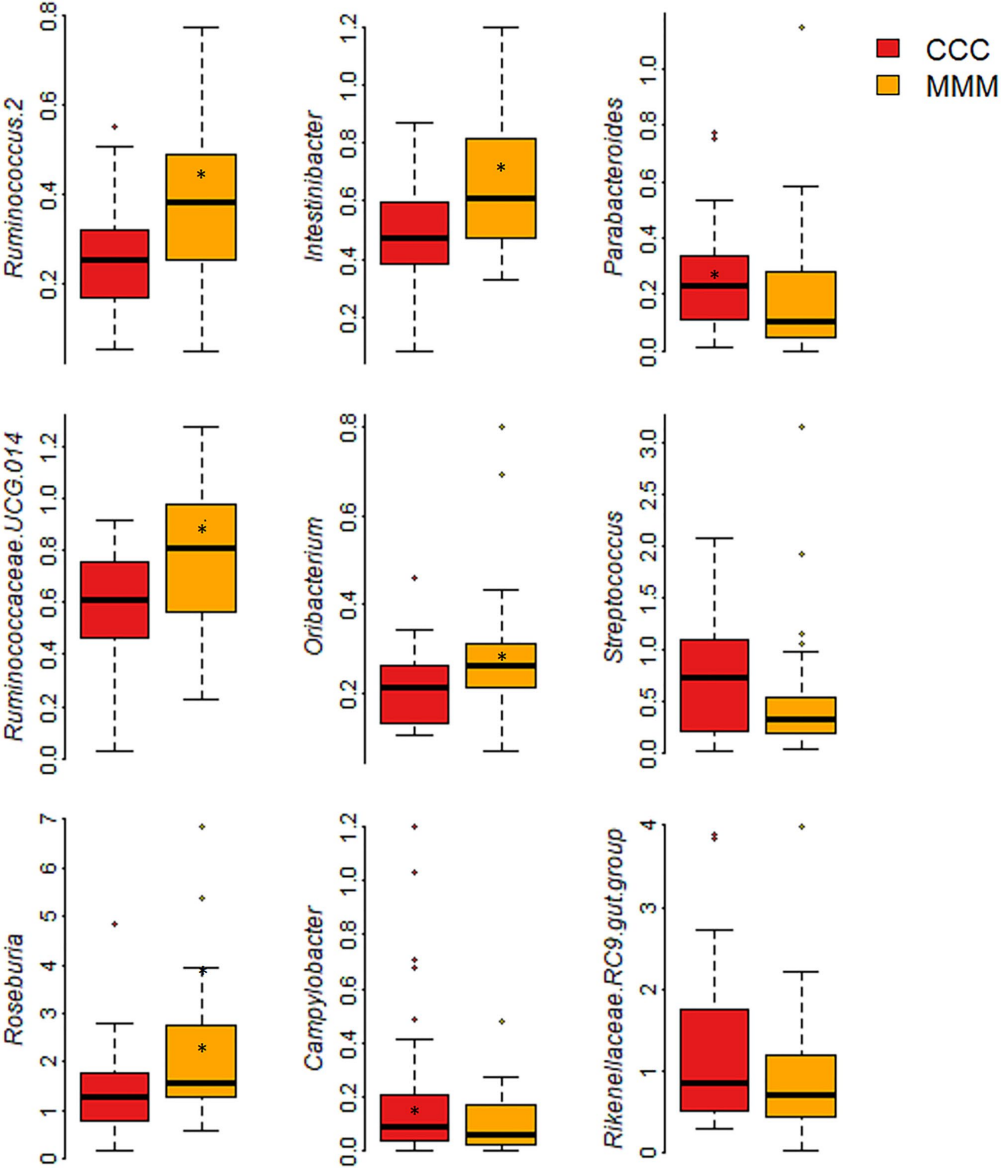
Relative abundances of microbiota between control (CCC), and MFC (MMM) piglet groups at genus level. Asterisks indicate significant differences (p < 0.05).

At genus level, *Ruminococcus.2, Ruminococcaceae.UCG.014, Intestinibacter*,*Roseburia,* and *Oribacterium* genera were highly abundant in the MFC group, whereas *Campylobacter* and *Parabacteroides* genera were abundant, and *Streptococcus* and the Rikenellaceae.RC9.gut.group tended to be abundant in the control group (p < 0.1) compared to levels in the MFC group (Fig. 5, Supplementary Table S1).

**Figure 6:**
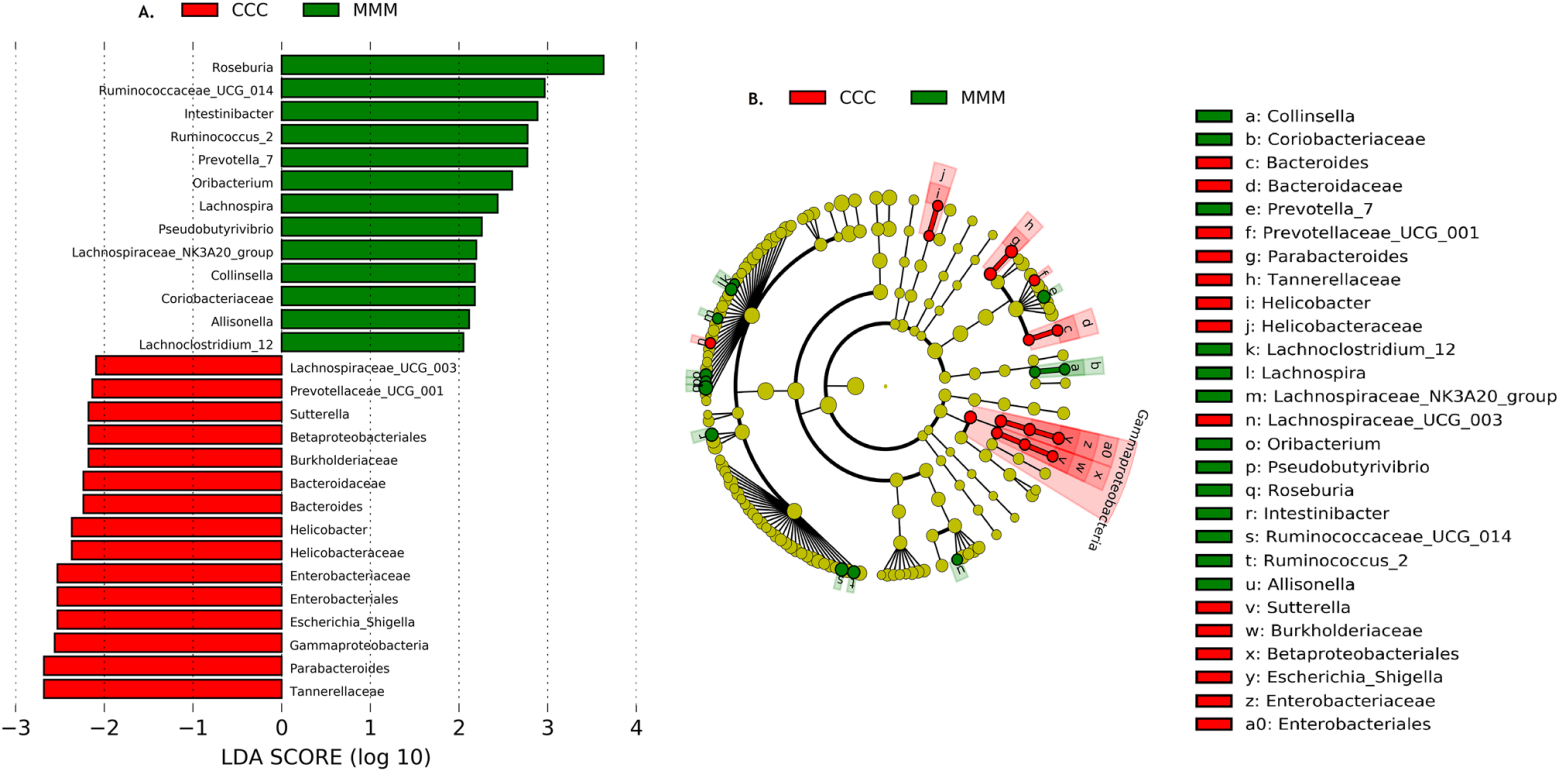
Linear discriminant analysis (LDA) effect size (LEfSe) of gut microbiota between control (CCC) and treatment (MMM) groups. (**A**) bar plot showing the differentially abundant taxa between groups, and (**B**) cladogram showing differences in abundant taxa between groups. LDA score threshold was > 2.0.

To further examine the differential abundant taxa, a LEFSe analysis with a LDA threshold > 2.0 was carried out at phylum-to-genus level. LEFSe analysis identified 28 taxa as potential biomarkers for the control group and MFC group; 15 taxa were unique to the control group, and 12 to the MFC group based on LDA score (Fig. 6).

At class level, Gammaproteobacteria was highly enriched in the control group. In addition, Betaproteobacteriales *and* Enterobacteriales were enriched in the control group at order level. *Bacteroidaceae*,*Burkholderiaceae, Enterobacteriaceae,* Helicobacteraceae*,* and Tannerellaceae families were more enriched in the control group than in the enriched Coriobacteriaceae family in the MFC group. At genus level, *Allisonella*,*Collinsella*,*Intestinibacter, Lachnoclostridium_12, Lachnospira*, the Lachnospiraceae_NK3A20_group, *Oribacterium, Prevotella_7, Pseudobutyrivibrio*,*Roseburia*, Ruminococcaceae_UCG_014, and *Ruminococcus_2* were highly enriched in the MFC group, whereas enrichment of Bacteroides*, Helicobacter,* Lachnospiraceae_UCG_003, *Parabacteroides*, Prevotellaceae _UCG_001, *Sutterella*, and *Escherichia_Shigella* genera occurred in the control group (Fig. 6).

We found some similarities between Mare and LEFSe analyses in regard to differential taxa abundance at class, order, family, and genus level (Table 3). The Gammaproteobacteria class and Tannerellaceae family were enriched in the control group in both analyses, whereas at genus level, *Ruminococcus.2, Roseburia*,*Intestinibacter*,*Oribacterium*, and *Ruminococcaceae.*UCG.014 genera were abundant in the MFC group. In PICRUSt2 functional analysis, we observed 247 enzymatic function, 627 KEGG orthology, and 98 molecular pathways were significant between groups at the OTU level. Top 20 pathways three analyses are visualized (Supplementary Fig. S1).

**Table 3:** Comparison of significant taxa based on relative abundance in Mare package, and estimated effect size (LEFSe) between control and MFC group.

Taxa	Mare package		LEFSe	
	CCC	MMM	CCC	MMM
Phylum	Epsilonbacteraeota			
Class	Campylobacteria Gammaproteobacteria		Gammaproteobacteria	
Order	Campylobacterales		Betaproteobacteriales Enterobacteriales	
Family	Campylobacteraceae Tannerellaceae Family_XIII Muribaculaceae		Enterobacteriaceae Tannerellaceae Burkholderiaceae Helicobacteraceae Bacteroidaceae	
Genus	*Campylobacter* *Parabacteroides*	*Ruminococcus.2* *Intestinibacter* *Roseburia* Ruminococcaceae.UCG.014 *Oribacterium*	*Bacteroides* *Helicobacter* *Escherichia_Shigella* Prevotellaceae_UCG_001 *Sutterella* *Parabacteroides* Lachnospiraceae_UCG_003	*Ruminococcus_2* *Intestinibacter* *Roseburia* *Pseudobutyrivibrio* Ruminococcaceae_UCG_014 *Oribacterium* *Lachnoclostridium_12* *Lachnospira* Lachnospiraceae_NK3A20_group *Prevotella_7* *Allisonella Collinsella*

*LEFSe* linear discriminant analysis effect size; *Control* CCC; *MFC* MMM; *CCC* sow control feed, piglets fed control creep feed, piglets post-weaning control feed; *MMM* sow MFC, piglets fed MFC creep feed, post-weaning piglets MFC feed.

### Correlations between microbial population and performance parameter

**Figure 7:**
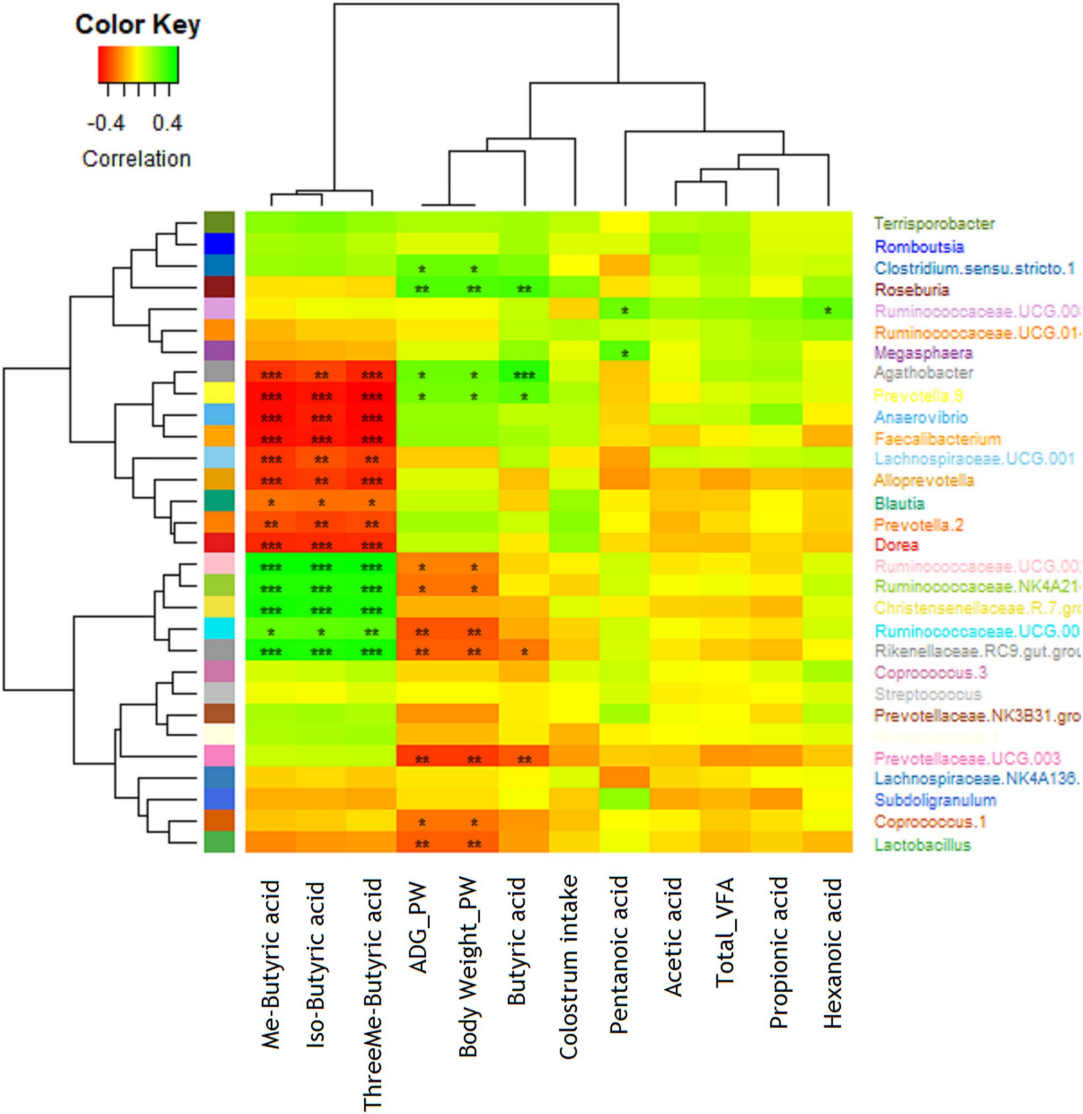
Correlations between microbial genera, and performance parameters and fecal volatile fatty acids (Total VFA, Acetic Acid, Propionic Acid, Butyric Acid, Isobutyric Acid, 2-MeButyric Acid, 3-MeButyric Acid, Pentanoic Acid, and Hexanoic Acid). The heatmap illustrates the positive (green) or negative (red) associations between microbial genera and parameters, with asterisks indicating significant differences (*p < 0.05).

*Clostridium.*sensu*.stricto.1, Roseburia*,*Agathobacter,* and *Prevotella.9* were positively correlated with ADG and body weight at 7 weeks of age. In addition, all the above genera except *Clostridium.*sensu*. stricto.1* were positively correlated with butyric acid (Fig. 7). However, Ruminococcaceae.UCG.014, Ruminococcaceae.NK4A21, Ruminococcaceae.UCG.002, Rikenellaceae.RC9.gut.group*,* Prevotellaceae.UCG.003*, Coprococcus.1*, and *Lactobacillus* were negatively correlated with ADG and body weight at age 7 weeks*. Agathobacter, Prevotella.9, Anaerovibrio*,*Faecalibacterium,* Lachnospiraceae.UCG.001, *Alloprevotella*,*Blautia, Prevotella.2*, and *Dorea* genera were negatively correlated with IsoButyric acid, 2-MeButyric acid, and 3-MeButyric acid. Ruminococcaceae.UCG.014, Ruminococcaceae.NK4A21, Ruminococcaceae.UCG.002, and Rikenellaceae.RC9.gut.group were positively correlated with IsoButyric acid, 2-MeButyric acid, and 3-MeButyric acid (Fig. 7).

## Discussion

In our study, MFC improved piglet performance both through maternal and dietary effects. The maternal effect was evidenced by better performance of those sows receiving MFC during suckling, whereas MFC diet directly improved growth performance of piglets after weaning. At post weaning, MFC supplementation reduced PWD incidence in piglets, raised the abundance of butyrate-producing beneficial bacteria, such as, such as, Ruminococcus.2, Ruminococcaceae.UCG.014, Intestinibacter, Roseburia, and Oribacterium genera and reduced in abundance pathogenic bacteria, such as Campylobacter, and Escherichia. In addition, piglets supplemented with MFC had higher bodyweight, and ADG at 3 weeks and at 7 weeks of age than did control piglets. Moreover, MFC supplementation tended to raise hexanoic acid and raise the butyric acid in the feces of post-weaning piglets.

As a management practice, providing creep feed is relevant to lactating piglets, because rooting and grazing behaviors are observable in wild pigs between 2 to 4 weeks of age^31,32^. A fibrous diet may play an essential role in preparing piglets for their post-weaning period by early development of their gastrointestinal tract. This may improve feed intake after weaning due to the adjustability of their diet, resulting in the maintenance of good intestinal health^31^. In our study, lactating piglets who had MFC effects from their mother, and had received MFC through creep feed, had higher ADG than did control piglets. This might be due to changes in their intestinal development, to improved intestinal permeability^31^, and to stimulation of VFA production by cellulose supplementation^33^. In this study, CMC group also had higher growth than control piglets, despite they only received MFC during their suckling period. Supplementation of dietary fiber in suckling piglets could affect their post-weaning performance by developing and stimulating their milk-oriented gut microbial population towards being fibrolytic-oriented, which would shift the microbiota that adapt the metabolic trait of breaking down the complex polysaccharides^34,35^. Dietary fiber in piglets promotes bacterial fermentation and production of VFA in the large intestine^36^ This VFA plays a crucial role in maintaining colonic health and acts as a key indicator of microbial fermentation in the intestine^37^.

We found, at post-weaning, that the butyrate level of piglets receiving MFC was significantly higher than that of control piglets. Butyrate plays a vital role in energy metabolism in the gut and in improving mucosal immunity^38^. In addition, butyrate can improve gut barrier function, the first-line defense against gut pathogens^39^, and can assist in maintaining a physical barrier by stimulating the goblet cells, followed by mucus secretion^40^. Butyrate, as a fuel for enterocytes, has the capacity to stimulate the growth of mucosa and stimulate cell differentiation, and also to improve barrier function^41,42^. Lower gut integrity, on the other hand, can lead to increased permeability to pathogens and to toxic metabolites^41^.

At post-weaning, piglets experience many types of physiological and environmental stress, which persist during the first weeks after weaning due to the changes in their diet, and changes in intestinal function followed by reduced feed consumption, low weight gain, indigestion, and diarrhea^43,44^. Dietary inclusion of insoluble fiber is shown to reduce piglets’ post-weaning diarrhea^45^. In our study, diarrhea incidence was reduced when MFC was supplemented to these post-weaning piglets. Dietary fiber, especially cellulose material, may block the attachment of gut pathogens, which will reduce their ability to stay in the gut, hence promoting their expulsion with the chyme and thereby reducing diarrhea incidence^46^. Dietary fiber may mitigate the gut microbial dysbiosis at weaning by creating an ambient environment for the growth of beneficial bacteria which reduce enterotoxigenic pathogenic bacteria, for example *E. coli*^33,47^. Moreover, fiber may encourage the functional maturation and growth of the gastrointestinal tract^43^. Due to its higher water-holding capacity, it may change the gastric transit through altering the rate of gastric emptying or altering gut motility in favor of gastrointestinal-tract (GIT) development^13^.

This development of GIT, especially in the large intestine, might be reducing diarrhea incidence by means of its high water-resorption capacity^31^. In addition, fibers like MFC may cause increased ileal nitrogen losses, which would facilitate the starch and protein as substrates for the gut microbiota; this is beneficial for the host^33,48^. We found that dietary inclusion of MFC after weaning reduced the incidence of PWD in post-weaning piglets. In other studies, supplementation of 1.5% cellulose reduced the incidence of PWD in such piglets^45,49^.

Another crucial mechanism by which MFC reduces PWD by MFC is by its ability to form extremely shear-thinning hydrogel with high water-binding capacity and with zero-shear viscosity. Which may facilitate tissue regeneration in injured intestinal layers, the same way that cellulosic hydrogel does in regeneration of bone, cartilage, and neural tissues^19^. Moreover, the end- product of MFC is glucose, which is beneficial for cell growth^50^.

At 3 and 7 weeks of age, piglets which received 2% MFC had significantly higher body weight than did control piglets. Several mechanisms may be responsible. First, this might be due to less energy loss through reduced diarrhea incidence; hence this energy could improve piglet body weight^51^. Another mechanism might be attributed to the high water-holding capacity of MFC, which results in the increased size of their digestive organs^52^.

Similarly, higher ADG was achieved by supplementation of 2% MFC to piglets after weaning, at age 3 weeks, and up until 7 weeks. Our findings corroborate those of Pascoal et al.^45^, who found higher ADG of post-weaning piglets after supplementation with 1.5% cellulose. These kinds of performance were also reported by other authors^53,54^, who observed improvements in weight gain and intestinal health by the inclusion in the diet of purified cellulose. Although Högberg et al.^52^ attributed such an improvement in weight gain to the increased size of the digestive organs, Gerritsen et al.^43^ suggest that this growth might be due to the better gut environment, resulting in high enzyme activity and microbiota modulation.

Piglets supplemented with 6% fibers of SBP origin showed no effect on ADG at their post-weaning, but at 5%, they might be able to raise ADG^55^. Supplementation with purified cellulose has been responsible for an increased villus height and crypt depth ratio, and decreased cox-2, which overcomes mucosal injury followed by piglets’ improved intestinal health^56^. In addition, Hanczakowska et al.^54^ demonstrated that a small amount of insoluble non-starch polysaccharide (iNSP) could improve piglets’ health and performance by making changes in gut morphology and gut pH, and by lowering the growth rate of pathogenic microbes.

It is well known that gut microbiota homeostasis plays a crucial role in the host’s gut health and immune organ maturation^57^. Gut microbial dysbiosis in piglets is related to many enteric diseases^2^, but stability of gut microbiota depends on feed supplementation and on the types of bacterial species, their abundance, and their interactions within the microbial community^58^. Effects of feed supplementation, especially of dietary fiber, are receiving increased attention due to their positive effects on enzymatic activities and the gut microbiota^43,59^.

Our study indicated that MFC supplementation modulated the gut microbiota of post-weaning piglets. It reduced the Epsilonbacteraeota phylum established by reclassification of the Epsilonproteobacteria and Desulfurellales^60^, widely known for containing several pathogenic genera, such as *Helicobacter, Arcobacter* and *Campylobacter*. However, MFC supplementation likely suppressed the abundance of Proteobacteria, and improved the abundance of Firmicutes. Member organisms of the Proteobacteria are gram-negative pathogenic bacteria including *Escherichia, Salmonella,* and *Vibrio,* regarded as being indicators of gut dysbiosis^61^, gut inflammation, and gut diseases^51^. This might be an explanation for the lower post-weaning diarrhea incidence in our MFC-supplemented piglets.

Members of the Firmicutes phylum are known for being butyrate producers, and this may have played a beneficial role in MFC piglets. Our study’s Campylobacteria and Gammaproteobacteria were suppressed by MFC supplementation. LEFSe analysis also revealed that MFC supplementation suppresses the enrichment of Gammaproteobacteria, Enterobacteriales, Enterobacteriaceae, and *Escherichia_Shigella*, a class-to-genus cluster. This taxonomic cluster is well known for its pathogenicity, as a few members of the Gammaproteobacteria—ones such as Escherichia—cause post-weaning diarrhea in piglets^62^. Some other genera are also associated with piglet diarrhea, for example, *Campylobacter*^63^. We established that MFC supplementation reduced the relative abundance of Campylobacteria, Campylobacterales, Campylobacteraceae, and *Campylobacter* at, respectively, class, order, family, and genus levels. *Escherichia* and *Campylobacter* can be regarded as etiologic agents of post-weaning diarrhea^62,64^, therefore explaining why MFC supplementation may be effective in lowering diarrhea incidence in post-weaning piglets.

Relative abundance of another pathogenic family, Tannerellaceae, was inhibited by the MFC supplementation. In human beings, organisms in the Tannerellaceae families are associated with oral infections called periodontitis^65^. Lachnospiraceae was likely higher in the MFC group. Members of this family associated with fiber degradation include cellulose and hemicellulose^66^. At genus level, *Ruminococcus.2, Ruminococcaceae.UCG.014, Roseburia, and Oribacterium* were elevated by MFC supplementation. Among them, *Ruminococcus* may degrade complex polysaccharides and produce butyrate^51^. In addition, *Roseburia* is well known for its beneficial effects on butyrate production, for stimulating the growth of beneficial bacteria, and for inhibiting the proliferation of pathogenic bacteria. Moreover, *Oribacterium* is also associated with butyrate production, and is linked with improved gut health and homeostasis^67^. Increased abundances of these butyrate genera: *Ruminococcus*, *Roseburia,* and *Oribacterium* in the MFC group may be connected with the increased butyrate production by MFC supplementation. Such supplementation may therefore not only promote ADG by providing energy for colonocytes, but also may improve gut health by exerting anti-inflammatory action^51^. A study conducted by Ju et al.^68^ revealed *Parabacteroides* to be associated with reduced body weight and ADG. In our study, the *Parabacteroides* genus was higher in the control group, whereas body weight and ADG were lower than in the MFC-supplemented piglets. This might explain why MFC piglets showed the dominant growth performance. Some of the *Streptococcus* genera of the Streptococcaceae family are less abundant in pigs with high feed efficiency^69^. In addition, a major pathogen, *Streptococcus suis,* is linked in pigs with high mortality and low performance^70^. In our study, MFC supplementation suppressed the growth of *Streptococcus*, which might have led to the higher body growth.

Our study has, however, some limitations. Because we included samples from CCC (as control group) and MMM (MFC group) piglets for volatile fatty-acid determination and microbial sequencing, we observed that piglets belonging to the CCC group showed inferior, and those in the MMM group showed superior growth performance. Another limitation of our study involved piglet numbers. Our sows were hyper-prolific and therefore unable to produce enough milk to feed all their piglets, so our practice was to include only 12 to 14 piglets per sow, and to exclude those piglets transferred to other nursing sows.

## Conclusions

Because our piglets supplemented with MFC had higher body weight and ADG than did control piglets, both pre- and post-weaning, and MFC supplementation led to raised butyrate content and to reduced diarrhea incidence in post-weaning piglets. In addition, MFC supplementation reduced the abundance of pathogenic bacteria and raised the abundance of butyrate-producing beneficial bacteria during post-weaning until 7 weeks of age. These beneficial effects attributable to the MFC supplementation at several stages of early development until 7 weeks. Considering all of this, it is promising that in piglets, MFC supplementation may be a potential feeding strategy to improve growth performance, microbial modulation, and volatile fatty acid content and to reduce post-weaning diarrhea.

## Materials and methods

### Statement

The experiment took place on a commercial farm in Kouvola, Finland, from February to April, 2021. We conducted the study according to the Declaration of Helsinki guidelines, and it was approved by the Southern Finland Regional State Administrative Agency ESAVI/2325/04.10.07/2017; modification ESAVI/17315/2020).

### Animals and sampling

#### Sow selection

Our study’s 45 multiparous sows Topigs Norsvin (TN 70) were balanced based on parity between treatment groups, and were selected based on the principle of first farrowing, first come from each group. Number of sows we selected by assuming that the farrowing duration is 260 min in treatment group and 285 min in the control group with a variance of 25 min. To find the desired difference, with a 80% power and 0.05 significance level. Allocation was considered complete when required number of sows was allocated in each group. During gestation, these sows were housed loose in groups of 10 to 15 in pens equipped with individual feeding stalls. From the last 5 weeks of farrowing, they were fed a standard gestation diet (Hankkija Oy, Hyvinkää, Finland) differing only in that 24 treatment sows received a daily 75 g MFC. After farrowing, both the control and treatment sows received a standard lactation diet (Supplementary Table S2) differing only in supplementation of 100 g MFC (Supplementary Table S3) per day/treatment sow until weaning (21 days).

### Pre-weaning piglet feeding

Half of the litters of MFC-treated sows received MFC (1%) -treated creep feed (Hankkija) (Supplementary Table S4), and the remaining litters received only control creep feed. In contrast, half the litters from the control sows received MFC (1%) -treated creep feed, and the remaining litters received only control creep feed (Fig. 1).

### Post-weaning Piglet Feeding

**Figure 8:**
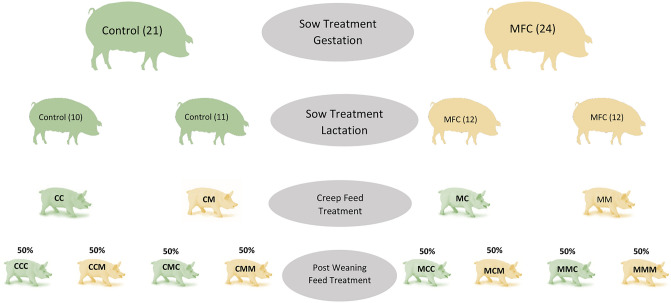
Schematic diagram of sow and piglet feeding plan. *MFC* Sows fed micro-fibrillated cellulose; *MM* sow MFC, piglets fed MFC creep feed; *MC* sow MFC, piglets fed control creep feed; *CM* sow control, piglets fed MFC creep feed; and CC sow control, piglets fed control creep feed. *MMM* sow MFC, piglets fed MFC creep feed, post-weaning piglets MFC feed; *MMC* sow MFC, piglets fed MFC creep feed, post-weaning piglets control feed; *MCM* sow MFC, piglets fed control creep feed, post-weaning MFC feed; *MCC* sow MFC, piglets fed control creep feed, post-weaning control feed; *CMM* sow control feed, piglets fed MFC creep feed, piglets post-weaning MFC feed; *CMC* sow control feed, piglets fed MFC creep feed, piglets post-weaning control feed; *CCM* sow control feed, piglets fed control creep feed, piglets post-weaning MFC feed; *CCC* sow control feed, piglets fed control creep feed, piglets post-weaning control feed. “Modified from Fig. 1. in Uddin et al., Animals.11, 2511 (2021)”.

At weaning, after 21 ± 1 days of lactation, 530 piglets from four pre-weaning piglet groups (MM = 155, MC = 135, CM = 109, and CC = 131) were included in the post-weaning feeding regime. Number of piglets were selected by assuming 7.8 kg piglets’ body weight at weaning in the treatment group and 7.5 kg in the control group with an equal variance of 0.7 and using 80% and 0.05 significance level. Each of the four pre-weaning piglet groups was divided equally, which produced eight treatment groups: CCC, CCM, CMC, CMM, MCC, MCM, MMC, and MMM. Whereas four treatment groups (CCM = 56, CMM = 54, MCM = 61, MMM = 77) received MFC (2%)—treated post-weaning feed (Hankkija) (Supplementary Table S4), the remaining four groups (CCC = 63, CMC = 48, MCC = 58, MMC = 74) received control post-weaning feed (Fig. 8). We followed the eight post-weaning treatment groups until they reached 7 weeks of age.

### Parameters and measurements

We calculate the colostrum intake by using a regression formula developed by Theil et al.^71^. We measured the body weight of these piglets at birth, at weaning (3 weeks of age), and at post-weaning (7 weeks) using a weighing scale (XL-Float-22, Patriot ®, Finland). We measured the average body weight (ADG) of piglets at weaning, and post-weaning by using following equation.

$$ {\text{ADG}} = \frac{{{\text{body\,weight}}\;{\text{of}}\;{\text{piglet}}\;{\text{at}}\;21\;{\text{or}}\;49\;{\text{days}}}}{{21\;or\;49}} $$

We calculated the piglets’ diarrhea incidence, mortality rate, and volatile fatty acids (VFA) at weaning and at post-weaning. At 24 h after birth, for management reasons, some piglets were allowed to move to other sows between days 2 to 21, their ear tags were removed, and these piglets we excluded from the study.

### Collection of fecal samples and diarrhea evaluation

On farrowing day, fresh fecal samples we collected from each sow’s rectum each into a sterile plastic bag (CON = 21, MFC = 24). We also collected piglets’ fecal samples at weaning (MM = 76, MC = 66, CM = 62, and CC = 67) and post-weaning (CCC = 36, CCM = 31, CMC = 29, CMM = 32, MCC = 33, MCM = 33, MMC = 36, and MMM = 40) into sterile bags and stored them at –80 °C for further analysis. During collection of these fecal samples, if the feces were liquid and not formed, the sample was considered diarrhea, and was counted in the calculation of diarrhea incidence.

### Volatile fatty acid (VFA) analysis

VFA of piglets’ (Total VFA, Acetic acid, Propionic acid, Butyric acid, IsoButyric acid, 2-MeButyric acid, 3-MeButyric acid, Pentanoic acid, and Hexanoic acid) feces at weaning and post-weaning we measured by ultra-performance liquid chromatography (UPLC). A fecal sample’s aliquot of 0.3 g was homogenized in 1.2 mL of distilled water and centrifuged at 18,000×*g* for 10 min at 4 °C. The resulting supernatant we filtered twice, first through a 0.45-µm syringe filter and then through a 0.22-µm syringe filter. The filtrate was stored at − 20 °C until analysis of VFA. Then the VFA content of the fecal samples of the piglets was determined by the method described by Puhakka et al^72^.

### Microbial characterization

We conducted DNA extraction by taking 250 mg of feces from each sample using the DNeasy PowerSoil Pro Kit (Qiagen, ct. no. 47014, Hilden, Germany) according to the manufacturer’s instructions. Then we quantified the yields and purity of the extracted DNA with Nanodrop 2000 (Thermo Fisher Scientific, Waltham, MA, USA). After that, we executed the 16S PCR amplification and sequencing with primer modifications by the method described by Pereira et al.^73^. After the amplification of the V3-V4 16s region, primers 341F_1–4 (CCTACGGGNGGCWGCAG) and 785R_1–4 (GACTACHVGGGTATCTAATCC) were added to the 5´ ends with partial Illumina TruSeq adapter sequences (Supplementary Table S5). Amplification of the PCR and Miseq sequencing we carried out by the method of Pereira et al.^73^. The whole process was executed at the DNA Sequencing and Genomics Laboratory, Institute of Biotechnology, University of Helsinki.

To pre-process the FASTQ files produced following Miseq sequencing steps, we used the Dada2 R package (https://benjjneb.github.io/dada2/tutorial_1_8.html). We truncated the forward reads at position 295, and at 160 for the reverse reads. To learn the error rate, we used the DADA2 algorithm followed by the dereplication step, which combines all the identical sequencing reads into unique sequences. Then we used dereplicated data to apply the algorithm of the core sample inference. In the following steps, to get the denoised sequences, we then merged the forward and the reverse reads by overlapping 20 bases where they were identical in the overlapped position. After constructing the sequence table, the dada method performed chimera removal. Then we looked at the number of reads in order to track whether any large drop occurred in any step of the pipeline. In the final steps, we assigned taxonomy to the sequences by using the Silva reference database. Then we organized the taxonomy for downstream analysis.

### Statistical analyses

We used Stata 17.0 (Stata MP/17 for Windows; Stata Corp., College Station, TX, USA) software for data analysis. In the descriptive statistics, we expressed data as Means ± standard error of mean (SEM) after running ANOVA. The significance level was considered P < 0.05, the tendency was considered between P > 0.05, and < 0.1. After getting significant effects between treatments, we compared means within treatments using functions pwcompare and mcompare (tukey).

For the microbiome analysis, we used the “Mare” package^74^ in R software (4.2.0). In downstream analysis, we measured the relative abundance (phylum to genus) by using the “GroupTest” function; alpha and beta diversity were estimated by use of R vegan package. Heatmap visualized the associations between microbiota and clinical variables using the R function of the mare package “CorrelationMap,” which implements Spearman’s correlation, and performed statistical tests at the different taxa levels. We estimated the Linear Discriminant Analysis (LDA) Effect Size (LEfSe) by using a galaxy computational tool (http://huttenhower.sph.harvard.edu/galaxy), following a metagenomic biomarker-discovery approach^75^. It performed differential abundance testing using the Kruskal–Wallis rank sum test between groups, and calculated the effect size at the 2.0 LDA score threshold. To determine the functional potential of the microbiota between groups, we used the Namco webtool (https://exbio.wzw.tum.de/namco/, accessed on 13th May 2022), which adapts the Phylogenetic Investigation of Communities by Reconstruction of Unobserved States (PICRUSt2)^76^ approach. It performs differential analysis based on the Kyoto Encyclopedia of Genes and Genomes (KEGG), number of enzymes classification, different Kyoto orthology (KO), and pathways by Aldex2^77^.

### Ethics approval and consent to participate

We followed all applicable guidelines and regulations in conducting our study. The use of live animals in this study was reviewed by the Animal Research Ethics Committee of the University of Helsinki, and approved by the Southern Finland Regional State Administrative Agency ESAVI/2325/04.10.07/2017; modification ESAVI/17315/2020). We carried out the study in compliance with the ARRIVE guidelines.

## Supplementary Information

Supplementary Figure S1.

Supplementary Tables.

## Data Availability

The datasets generated and/or analysed during the current study are available in the Sequence Read Archive repository, in the BioProject PRJNA857074.

## References

[1] Campbell JM, Crenshaw JD, Polo J (2013). The biological stress of early weaned piglets. J. Anim. Sci. Biotechnol..

[2] Gresse R (2017). Gut microbiota dysbiosis in postweaning piglets: Understanding the keys to health. Trends Microbiol..

[3] Hasan S (2019). Late gestation diet supplementation of resin acid-enriched composition increases sow colostrum immunoglobulin G content, piglet colostrum intake and improve sow gut microbiota. Animal.

[4] Jha R, Rossnagel B, Pieper R, Van Kessel A, Leterme P (2010). Barley and oat cultivars with diverse carbohydrate composition alter ileal and total tract nutrient digestibility and fermentation metabolites in weaned piglets. Animal.

[5] Metzler B, Mosenthin R (2008). A review of interactions between dietary fiber and the gastrointestinal microbiota and their consequences on intestinal phosphorus metabolism in growing pigs. Asian-Aust. J. Anim. Sci..

[6] Williams BA, Verstegen MW, Tamminga S (2001). Fermentation in the large intestine of single-stomached animals and its relationship to animal health. Nutr. Res. Rev..

[7] Shang Q, Liu S, Liu H, Mahfuz S, Piao X (2021). Impact of sugar beet pulp and wheat bran on serum biochemical profile, inflammatory responses and gut microbiota in sows during late gestation and lactation. J. Anim. Sci. Biotechnol..

[8] Bergman EJ (1990). Energy contributions of volatile fatty acids from the gastrointestinal tract in various species. Physiol. Rev..

[9] Yen J, Nienaber J, Hill D, Pond WJ (1991). Potential contribution of absorbed volatile fatty acids to whole-animal energy requirement in conscious swine. J. Anim. Sci..

[10] Varel V, Yen JTJ (1997). Microbial perspective on fiber utilization by swine. J. Anim. Sci..

[11] Koh A, De Vadder F, Kovatcheva-Datchary P, Bäckhed F (2016). From dietary fiber to host physiology: Short-chain fatty acids as key bacterial metabolites. Cell.

[12] Kreuzer M (1998). Reduction of gaseous nitrogen loss from pig manure using feeds rich in easily-fermentable non-starch polysaccharides. Anim. Feed Sci. Technol..

[13] Molist F (2014). Relevance of functional properties of dietary fibre in diets for weanling pigs. Anim. Feed Sci. Technol..

[14] Konstantinov SR (2003). Effect of fermentable carbohydrates on piglet faecal bacterial communities as revealed by denaturing gradient gel electrophoresis analysis of 16S ribosomal DNA. FEMS Microbiol. Ecol..

[15] Wu R, Zhang H, Zeng X, Zhang J, Xiong H (2011). L-Arabinose and oligosaccharides production from sugar beet pulp by xylanase and acid hydrolysis. Afr. J. Biotech..

[16] Von Heimendahl E, Breves G, Abel H (2010). Fiber-related digestive processes in three different breeds of pigs. J. Anim. Sci..

[17] Serpa A (2016). Vegetable nanocellulose in food science: A review. Food Hydrocolloids.

[18] Ciolacu DE, Nicu R, Ciolacu F (2020). Cellulose-based hydrogels as sustained drug-delivery systems. Materials.

[19] Leone G, Fini M, Torricelli P, Giardino R, Barbucci R (2008). An amidated carboxymethylcellulose hydrogel for cartilage regeneration. J. Mater. Sci. Mater. Med..

[20] Le Sciellour M, Labussière E, Zemb O, Renaudeau D (2018). Effect of dietary fiber content on nutrient digestibility and fecal microbiota composition in growing-finishing pigs. PLoS ONE.

[21] Chen X, Xu J, Ren E, Su Y, Zhu W (2018). Co-occurrence of early gut colonization in neonatal piglets with microbiota in the maternal and surrounding delivery environments. Anaerobe.

[22] Holman DB, Chénier MR (2014). Temporal changes and the effect of subtherapeutic concentrations of antibiotics in the gut microbiota of swine. FEMS Microbiol. Ecol..

[23] Le Floc’h N (2014). Impact of feed restriction on health, digestion and faecal microbiota of growing pigs housed in good or poor hygiene conditions. Animal.

[24] Heinritz SN (2016). Intestinal microbiota and microbial metabolites are changed in a pig model fed a high-fat/low-fiber or a low-fat/high-fiber diet. PLoS ONE.

[25] Verschuren LM (2018). Fecal microbial composition associated with variation in feed efficiency in pigs depends on diet and sex. J. Anim. Sci..

[26] Desai MS (2016). A dietary fiber-deprived gut microbiota degrades the colonic mucus barrier and enhances pathogen susceptibility. Cell.

[27] Lizardo R, Aumaître A (2001). Non-starch polysaccharides of sugar-beet pulp improve the adaptation to the starter diet, growth and digestive process of the weaned pig. Cahiers Opt. Méditerr..

[28] Hermes R (2010). Effects of type of cereal and fibre level on growth and parameters of the gastrointestinal tract in young pigs. Livest. Sci..

[29] Laitat M (2015). Influence of sugar beet pulp on feeding behavior, growth performance, carcass quality and gut health of fattening pigs. Biotechnol. Agron. Soc. Environ..

[30] Yan C (2017). Effect of dietary sugar beet pulp supplementation on growth performance, nutrient digestibility, fecal microflora, blood profiles and diarrhea incidence in weaning pigs. J. Anim. Sci. Technol..

[31] van Hees H (2021). Fibre supplementation to pre-weaning piglet diets did not improve the resilience towards a post-weaning enterotoxigenic *E. coli* challenge. J. Anim. Physiol. Anim. Nutr..

[32] Petersen V (1994). The development of feeding and investigatory behaviour in free-ranging domestic pigs during their first 18 weeks of life. Appl. Anim. Behav. Sci..

[33] Van Hees H (2019). Dietary fibre enrichment of supplemental feed modulates the development of the intestinal tract in suckling piglets. J. Anim. Sci. Biotechnol..

[34] Choudhury R (2021). Early life feeding accelerates gut microbiome maturation and suppresses acute post-weaning stress in piglets. Environ. Microbiol..

[35] Frese SA, Parker K, Calvert CC, Mills DA (2015). Diet shapes the gut microbiome of pigs during nursing and weaning. Microbiome.

[36] Metzler-Zebeli BU, Zijlstra RT, Mosenthin R, Gänzle MG (2011). Dietary calcium phosphate content and oat β-glucan influence gastrointestinal microbiota, butyrate-producing bacteria and butyrate fermentation in weaned pigs. FEMS Microbiol. Ecol..

[37] Mu C, Zhang L, He X, Smidt H, Zhu W (2017). Dietary fibres modulate the composition and activity of butyrate-producing bacteria in the large intestine of suckling piglets. Antonie Van Leeuwenhoek.

[38] Tremaroli V, Bäckhed F (2012). Functional interactions between the gut microbiota and host metabolism. Nature.

[39] Zheng L (2017). Microbial-derived butyrate promotes epithelial barrier function through IL-10 receptor–dependent repression of claudin-2. J. Immunol..

[40] Wrzosek L (2013). Bacteroides thetaiotaomicron and Faecalibacterium prausnitzii influence the production of mucus glycans and the development of goblet cells in the colonic epithelium of a gnotobiotic model rodent. BMC Biol..

[41] Scheppach W (1994). Effects of short chain fatty acids on gut morphology and function. Gut.

[42] Le Gall M (2009). Comparative effect of orally administered sodium butyrate before or after weaning on growth and several indices of gastrointestinal biology of piglets. Br. J. Nutr..

[43] Gerritsen R, van Der Aar P, Molist F (2012). Insoluble nonstarch polysaccharides in diets for weaned piglets. J. Anim. Sci..

[44] Lallès J-P, Bosi P, Smidt H, Stokes CR (2007). Weaning: A challenge to gut physiologists. Livest. Sci..

[45] Pascoal LAF (2012). Fiber sources in diets for newly weaned piglets. Rev. Bras. Zootecnia.

[46] Schley P, Field C (2002). The immune-enhancing effects of dietary fibres and prebiotics. Br. J. Nutr..

[47] Schokker D (2018). Supplementation of fructooligosaccharides to suckling piglets affects intestinal microbiota colonization and immune development. J. Anim. Sci..

[48] Schulze H, Van Leeuwen P, Verstegen M, Van den Berg J (1995). Dietary level and source of neutral detergent fiber and ileal endogenous nitrogen flow in pigs. J. Anim. Sci..

[49] Flis M, Sobotka W, Antoszkiewicz Z (2017). Fiber substrates in the nutrition of weaned piglets: A review. Ann. Anim. Sci..

[50] Sannino A, Demitri C, Madaghiele M (2009). Biodegradable cellulose-based hydrogels: Design and applications. Materials.

[51] Uddin MK, Hasan S, Mahmud M, Peltoniemi O, Oliviero C (2021). In-feed supplementation of resin acid-enriched composition modulates gut microbiota, improves growth performance, and reduces post-weaning diarrhea and gut inflammation in piglets. Animals.

[52] Högberg A, Lindberg JE (2004). Influence of cereal non-starch polysaccharides and enzyme supplementation on digestion site and gut environment in weaned piglets. Anim. Feed Sci. Technol..

[53] Han Y, Han K, Lee J (2005). Effects of insoluble dietary fiber supplementation on performance and nutrient digestibility of weanling pigs. J. Anim. Sci. Technol..

[54] Hanczakowska E, Swiatkiewicz M, Bialecka A (2008). Pure cellulose as feed supplement for piglets. Medycyna Wet.

[55] Jeaurond E, Rademacher M, Pluske J, Zhu C, De Lange C (2008). Impact of feeding fermentable proteins and carbohydrates on growth performance, gut health and gastrointestinal function of newly weaned pigs. Can. J. Anim. Sci..

[56] Cho HM, Kim E, Wickramasuriya SS, Shin TK, Heo JM (2021). Growth and gut performance of young pigs in response to different dietary cellulose concentration and rearing condition. Asian-Australas. J. Anim. Sci..

[57] Sommer F, Anderson JM, Bharti R, Raes J, Rosenstiel P (2017). The resilience of the intestinal microbiota influences health and disease. Nat. Rev. Microbiol..

[58] Coyte KZ, Schluter J, Foster KR (2015). The ecology of the microbiome: Networks, competition, and stability. Science.

[59] Molist F, Manzanilla EG, Pérez JF, Nyachoti CM (2012). Coarse, but not finely ground, dietary fibre increases intestinal Firmicutes: Bacteroidetes ratio and reduces diarrhoea induced by experimental infection in piglets. Br. J. Nutr..

[60] Waite DW (2017). Comparative genomic analysis of the class Epsilonproteobacteria and proposed reclassification to Epsilonbacteraeota (phyl nov). Front. Microbiol..

[61] Shin N-R, Whon TW, Bae J-W (2015). Proteobacteria: Microbial signature of dysbiosis in gut microbiota. Trends Biotechnol..

[62] Luppi A (2016). Prevalence of virulence factors in enterotoxigenic Escherichia coli isolated from pigs with post-weaning diarrhoea in Europe. Porcine Health Manag..

[63] Cremonesi P (2022). Gut microbiome modifications over time when removing in-feed antibiotics from the prophylaxis of post-weaning diarrhea in piglets. PLoS ONE.

[64] Adhikari B, Kim SW, Kwon YM (2019). Characterization of microbiota associated with digesta and mucosa in different regions of gastrointestinal tract of nursery pigs. Int. J. Mol. Sci..

[65] Jensen A, LadegaardGrønkjær L, Holmstrup P, Vilstrup H, Kilian M (2018). Unique subgingival microbiota associated with periodontitis in cirrhosis patients. Sci. Rep..

[66] Biddle A, Stewart L, Blanchard J, Leschine S (2013). Untangling the genetic basis of fibrolytic specialization by Lachnospiraceae and Ruminococcaceae in diverse gut communities. Diversity.

[67] McCormack UM (2018). Fecal microbiota transplantation in gestating sows and neonatal offspring alters lifetime intestinal microbiota and growth in offspring. MSystems.

[68] Oh JK (2020). Association between the body weight of growing pigs and the functional capacity of their gut microbiota. Anim. Sci. J..

[69] McCormack UM (2017). Exploring a possible link between the intestinal microbiota and feed efficiency in pigs. Appl. Environ. Microbiol..

[70] Shao Y (2020). Differences in gut microbial and serum biochemical indices between sows with different productive capacities during perinatal period. Front. Microbiol..

[71] Theil P (2014). Mechanistic model to predict colostrum intake based on deuterium oxide dilution technique data and impact of gestation and prefarrowing diets on piglet intake and sow yield of colostrum. J. Anim. Sci..

[72] Puhakka L, Jaakkola S, Simpura I, Kokkonen T, Vanhatalo A (2016). Effects of replacing rapeseed meal with fava bean at 2 concentrate crude protein levels on feed intake, nutrient digestion, and milk production in cows fed grass silage–based diets. J. Dairy Sci..

[73] Pereira PA (2017). Oral and nasal microbiota in Parkinson's disease. Parkinsonism Relat. Disord..

[74] Korpela, K. *Mare: Microbiota Analysis in R Easily. R Package Version 1, 10.5281* (2016).

[75] Segata N (2011). Metagenomic biomarker discovery and explanation. Genome Biol..

[76] Douglas GM (2020). PICRUSt2 for prediction of metagenome functions. Nat. Biotechnol..

[77] Fernandes AD, Macklaim JM, Linn TG, Reid G, Gloor GB (2013). ANOVA-like differential expression (ALDEx) analysis for mixed population RNA-Seq. PLoS ONE.

