# Supplementary figures and images for: Dietary micro-fibrillated cellulose improves growth, reduces diarrhea, modulates gut microbiota, and increases butyrate production in post-weaning piglets
Source: Sci Rep. 2023 Apr 16;13:6194. doi: 10.1038/s41598-023-33291-z (PMC10106463; doi:10.1038/s41598-023-33291-z)

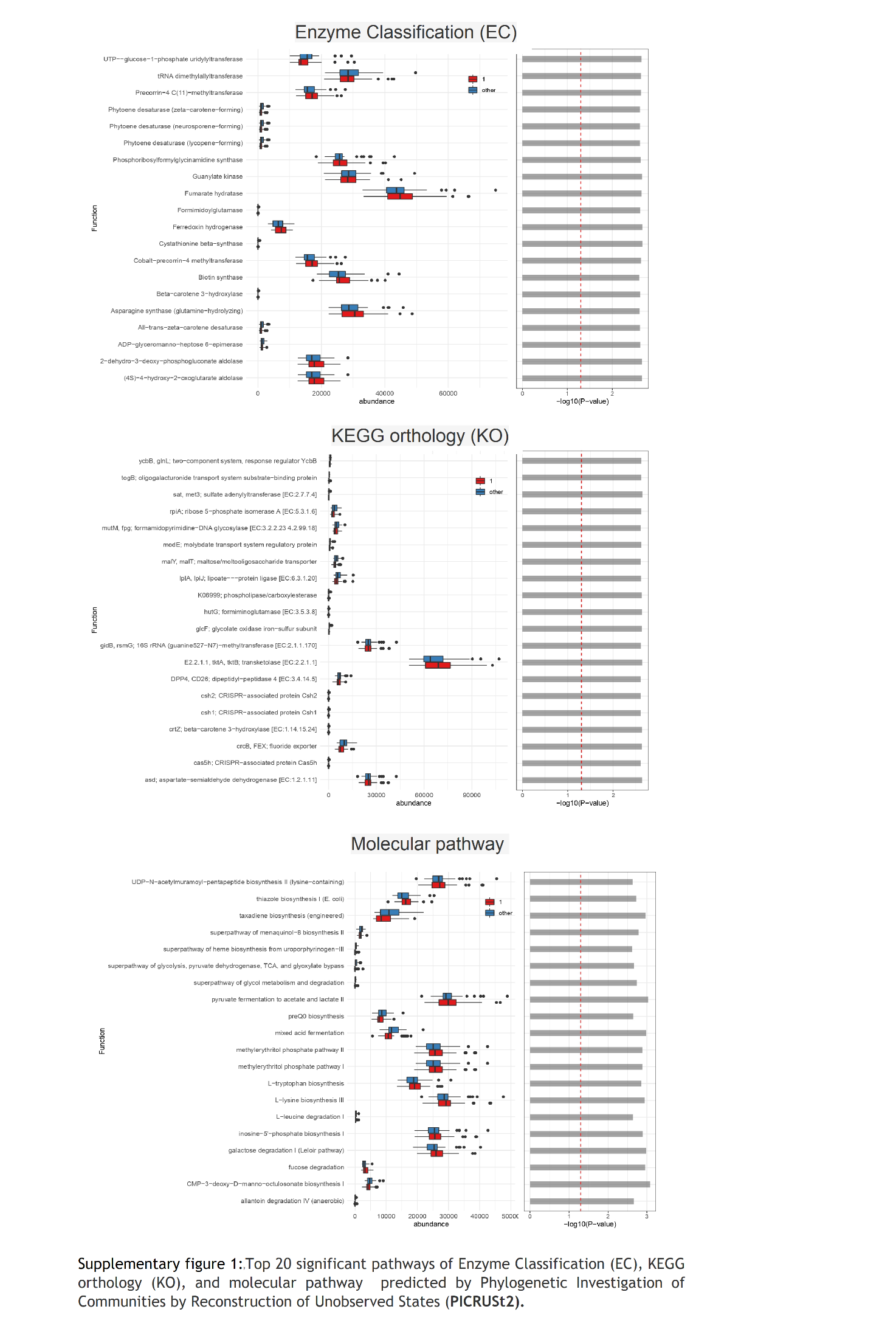

Supplement: Supplementary file 1 — Supplementary Figure S1. [file 41598_2023_33291_MOESM1_ESM.png]
