# Supplementary material for: Dietary micro-fibrillated cellulose improves growth, reduces diarrhea, modulates gut microbiota, and increases butyrate production in post-weaning piglets
Source: Sci Rep. 2023 Apr 16;13:6194. doi: 10.1038/s41598-023-33291-z (PMC10106463; doi:10.1038/s41598-023-33291-z)
Supplement: Supplementary file 2 — Supplementary Tables. [file 41598_2023_33291_MOESM2_ESM.docx]

**Dietary micro-fibrillated cellulose improves growth, reduces diarrhea, modulates gut microbiota, and increases butyrate production in post-weaning piglets**

Md Karim Uddin^1*^, Md. Rayhan Mahmud^1^, Shah Hasan^1^, Olli Peltoniemi^1^, and Claudio Oliviero^1^

^1^Department of Production Animal Medicine, Faculty of Veterinary Medicine, University of Helsinki, Finland

*Correspondence: md.uddin@helsinki.fi

**Supplementary Table S1.** Abundant taxa found between control (CCC), and treatment (MMM) groups using “mare” package of R.

| Taxa | CCC | MMM | Parameter_estimate | P-Value |
| --- | --- | --- | --- | --- |
| **Phylum** |  |  |  |  |
| Epsilonbacteraeota | 0.002 | 0.001 | -0.820 | **0.005** |
| Proteobacteria | 0.008 | 0.006 | -0.002 | 0.111 |
| Firmicutes | 0.813 | 0.827 | 0.018 | 0.139 |
| **Class** |  |  |  |  |
| Campylobacteria | 0.002 | 0.001 | -0.820 | **0.005** |
| Gammaproteobacteria | 0.003 | 0.002 | -0.421 | **0.053** |
| Clostridia | 0.678 | 0.720 | 0.042 | 0.082 |
| Bacilli | 0.089 | 0.063 | -0.473 | 0.098 |
| **Order** |  |  |  |  |
| Campylobacterales | 0.002 | 0.001 | -0.820 | **0.005** |
| Clostridiales | 0.678 | 0.720 | 0.042 | 0.082 |
| Lactobacillales | 0.089 | 0.063 | -0.473 | 0.098 |
| **Family** |  |  |  |  |
| Campylobacteraceae | 0.002 | 0.001 | -0.720 | **0.015** |
| Tannerellaceae | 0.003 | 0.002 | -0.756 | **0.022** |
| Family_XIII | 0.009 | 0.007 | -0.002 | **0.039** |
| Muribaculaceae | 0.012 | 0.009 | -0.254 | **0.049** |
| Streptococcaceae | 0.007 | 0.005 | -0.441 | 0.065 |
| Rikenellaceae | 0.013 | 0.009 | -0.372 | 0.074 |
| Lachnospiraceae | 0.209 | 0.228 | 0.019 | 0.134 |
| **Genus** |  |  |  |  |
| *Ruminococcus.2* | 0.003 | 0.004 | 0.380 | **0.003** |
| *Intestinibacter* | 0.005 | 0.006 | 0.263 | **0.003** |
| *Roseburia* | 0.014 | 0.021 | 0.385 | **0.009** |
| *Campylobacter* | 0.002 | 0.001 | -0.720 | **0.015** |
| Ruminococcaceae.UCG.014 | 0.006 | 0.008 | 0.271 | **0.016** |
| *Oribacterium* | 0.002 | 0.003 | 0.001 | **0.018** |
| *Parabacteroides* | 0.003 | 0.002 | -0.756 | **0.022** |
| *Streptococcus* | 0.007 | 0.005 | -0.441 | 0.065 |
| Rikenellaceae.RC9.gut.group | 0.013 | 0.009 | -0.377 | 0.068 |

**Supplementary Table S2.** Nutrient, and chemical composition of sow gestation, and lactation diet

| **Nutrient composition** | |  | |
| --- | --- | --- | --- |
| **Gestation diet** | | **Lactation diet** | |
| **Control** | **MFC** | **Control** | **MFC** |
| Barley 29.95 | Barley 29.95 | Dehulled oats 25.44 | Dehulled oats 25.44 |
| Oats 14.00 | Oats 14.00 | Barley 16.30 | Barley 16.30 |
| Wheat bran 14.00 | Wheat bran 14.00 | Wheat 15.00 | Wheat 15.00 |
| Wheat 7.00 | Wheat 7.00 | Soyabean meal 11.10 | Soyabean meal 11.10 |
| Sugar beet pulp 6.60 | Dehulled oats 6.00 | Oats 7.00 | Oats 7.00 |
| Dehulled oats 6.00 | Wheat feed meal 5.00 | Wheat bran 6.50 | Wheat bran 6.50 |
| Wheat feed meal 5.00 | Oat hulls 4.40 | Wheat feed meal 3.00 | Wheat feed meal 3.00 |
| Oat hulls 4.40 | Sugar beet pulp 4.10 | Vegetable oils and fatty acids 2.10 | Vegetable oils and fatty acids 2.10 |
| Sunflower seed meal 3.00 | Sunflower seed meal 3.00 | Oat hulls 2.00 | Oat hulls 2.00 |
| Peas 2.00 | MFC-fibre 2.50 | Sugar beet pulp 2.00 | Sugar beet pulp 2.00 |
| Soyabean meal 1.60 | Peas 2.00 | Calcium carbonate 1.74 | Calcium carbonate 1.74 |
| Calcium carbonate 1.33 | Soyabean meal 1.60 | Premixes 1.09 | Premixes 1.09 |
| Vegetable oils and fatty acids 1.30 | Calcium carbonate 1.33 | Faba bean (Horse bean) 1.00 | Faba bean (Horse bean) 1.00 |
| Molasses 1.00 | Vegetable oils and fatty acids 1.30 | Linseed expeller 1.00 | Linseed expeller 1.00 |
| Progut. yeast hydrolysate 0.95 | Molasses 1.00 | Glucose 1.00 | Glucose 1.00 |
| Premixes 0.79 | Progut. yeast hydrolysate 0.95 | Peas 1.00 | Peas 1.00 |
| Sodium bicarbonate 0.48 | Premixes 0.79 | Progut. yeast hydrolysate 0.95 | Progut. yeast hydrolysate 0.95 |
| Amino acids 0.28 | Sodium bicarbonate 0.48 | Amino acids 0.68 | Amino acids 0.68 |
| Salt 0.19 | Amino acids 0.28 | Salt 0.51 | Salt 0.51 |
| Mycotoxin binder 0.10 | Salt 0.19 | Defluorinated monocalcium phosphate 0.44 | Defluorinated monocalcium phosphate 0.44 |
| Defluorinated monocalcium phosphate 0.03 | Mycotoxin binder 0.10 | Progres. tall oil fatty acids 0.10 | Progres. tall oil fatty acids 0.10 |
|  | Defluorinated monocalcium phosphate 0.03 | Magnesium oxide 0.05 | Magnesium oxide 0.05 |

| **Chemical composition** | | | | |
| --- | --- | --- | --- | --- |
|  | **Gestation diet** | | **Lactation diet** | |
| Analysis (per kg) | Control | MFC | Control | MFC |
| DM (g) | 87,5 | 87,5 | 87,5 | 87,5 |
| Ash (g) | 5,5 | 5,5 | 5,9 | 5,9 |
| Crude protein (g) | 13,0 | 13,0 | 15,8 | 15,8 |
| Crude fat (g) | 4,4 | 4,4 | 5,7 | 5,7 |
| Crude fiber (g) | 7,8 | 7,8 | 5,4 | 5,4 |
| Lysine (g) | 6,1 | 6,1 | 9,8 | 9,8 |
| Methionine (g) | 2,1 | 2,1 | 3,0 | 3,0 |
| Calcium (g) | 6,8 | 6,8 | 9,8 | 9,8 |
| Phosphorus (g) | 4,8 | 4,8 | 5,3 | 5,3 |
| Sodium (g) | 2,4 | 2,4 | 2,2 | 2,2 |
| NE (MJ) | 8,8 | 8,8 | 9,9 | 9,9 |

**Supplementary Table S3. Composition and nutritive value of MFC**

| Analysis (per kg) | amount |
| --- | --- |
| Moisture,% | 8.2 |
| Dry matter,% | 91.8 |
| Crude protein (N * 6.25),g/kg ka | 95 |
| Crude fat,g/kg ka | 28 |
| Crude fiber,g/kg ka | 205 |
| NDF fiber,g/kg ka | 439 |
| Ash,g/kg ka | 84 |
| Nitrogen-free extracts,g/kg ka | 588 |
| Phosphorus (P),g/kg ka | 0.61 |
| Potassium (K),g/kg ka | 2.4 |
| Calcium (Ca),g/kg ka | 7.8 |
| Magnesium (Mg),g/kg ka | 2.4 |
| Sodium (Na),g/kg ka | 15 |
| Sulfur (S),g/kg ka | 1.7 |
| Iron (Fe),mg/kg ka | 310 |
| Copper (Cu),mg/kg ka | <5.2 |
| Manganese (Mn),mg/kg ka | 54 |
| Zinc (Zn),mg/kg ka | <21 |
| K / (Ca + Mg) eq ratio, | 0.11 |
| Selenium (Se),mg/kg ka | 0.10 |
| Chlorides (Cl-),g/100 g | <0.05 |
| Chloride for table salt,g/100 g | <0.10 |
| NaCl,µg/100 g | <21 (LOQ) |
| Vitamin D3,µg/100 g | <0.25 (LOQ) |
| (colecalciferol),mg/100 g | 1.15 |
| alpha-tocopherol (vitamin E),% ka | <0.5 (LOD) |

**Supplementary Table S4.** Nutrient, and chemical composition of piglets’ creep feed, post-weaning feed.

| Nutrient composition | | | | | | | | | | | |
| --- | --- | --- | --- | --- | --- | --- | --- | --- | --- | --- | --- |
| **Pre-weaning creep feed** | | | **Post-weaning feed** | | | | | | | | |
| **(7-22)** | | | **23-35 d** | | | | | **35-49 d** | | | |
| **Control** | **MFC** | | **Control** | | | **MFC** | | **Control** | | **MFC** | |
| Dehulled oats 21.62 | Dehulled oats 21.62 | | Barley 30.0 | | | Barley 30.0 | | Barley 30.00 | | Barley 30.00 | |
| Wheat 20.00 | Wheat 20.00 | | Dehulled oats 15.94 | | | Dehulled oats 15.94 | | Wheat 20.30 | | Wheat 20.30 | |
| Barley 13.40 | Barley 13.30 | | Wheat 14.60 | | | Wheat 14.60 | | Dehulled oats 18.04 | | Dehulled oats 18.04 | |
| Steamed corn 10.00 | Steamed corn 10.00 | | Whey powder 5.28 | | | Whey powder 5.28 | | Oats 6.30 | | Oats 6.30 | |
| Skimmed milk powder 6.64 | Skimmed milk powder 6.64 | | Steamed corn 5.00 | | | Steamed corn 5.00 | | Wheat bran 5.00 | | Wheat bran 5.00 | |
| Whey meal 5.38 | Whey meal 5.38 | | Wheat bran 4.00 | | | Wheat bran 4.00 | | Soyabean meal 3.10 | | Soyabean meal 3.10 | |
| Oats 3.50 | Oats 3.50 | | Oats 3.60 | | | Oats 3.60 | | Premixes 2.56 | | Premixes 2.56 | |
| Premixes 3.08 | Premixes 3.08 | | Premixes 3.50 | | | Premixes 3.50 | | Soya protein concentrate 2.03 | | Soya protein concentrate 2.03 | |
| Fish meal 2.50 | Fish meal 2.50 | | Oat hulls 3.00 | | | Oat hulls 3.00 | | Potato protein 2.00 | | Potato protein 2.00 | |
| Coconut oil 1.95 | Coconut oil 1.95 | | Skimmed milk powder 2.64 | | | Skimmed milk powder 2.64 | | Whey powder 1.48 | | Whey powder 1.48 | |
| Wheat bran 1.90 | Wheat gluten 1.51 | | Potato protein 2.16 | | | Potato protein 2.16 | | Linseed expeller 1.30 | | Linseed expeller 1.30 | |
| Wheat gluten 1.51 | Amino acids 1.50 | | Soya protein concentrate 2.12 | | | Soya protein concentrate 2.12 | | Amino acids 1.18 | | Amino acids 1.18 | |
| Amino acids 1.50 | Linseed expeller 1.50 | | Linseed expeller 1.30 | | | Linseed expeller 1.30 | | Glucose 1.02 | | Glucose 1.02 | |
| Linseed expeller 1.50 | Potato protein 1.37 | | Vegetable oils and fatty acids 1.25 | | | Vegetable oils and fatty acids 1.25 | | Fish meal 1.00 | | Fish meal 1.00 | |
| Potato protein 1.37 | Glucose 1.17 | | Glucose 1.07 | | | Glucose 1.07 | | Vegetable oils and fatty acids 1.00 | | Vegetable oils and fatty acids 1.00 | |
| Glucose 1.17 | Soya protein concentrate 1.10 | | Fish meal 1.00 | | | Fish meal 1.00 | | Progut. yeast hydrolysate 0.96 | | Progut. yeast hydrolysate 0.96 | |
| Soya protein concentrate 1.10 | MFC-fibre 1.00 | | Amino acids 0.90 | | | Amino acids 0.90 | | Calcium carbonate 0.76 | | Calcium carbonate 0.76 | |
| Vegetable oils and fatty acids 0.55 | Wheat bran 1.00 | | Coconut oil 0.77 | | | Coconut oil 0.77 | | Skimmed milk powder 0.72 | | Skimmed milk powder 0.72 | |
| Salt 0.46 | Vegetable oils and fatty acids 0.55 | | Wheat gluten 0.60 | | | Wheat gluten 0.60 | | Salt 0.46 | | Salt 0.46 | |
| Progut. yeast hydrolysate 0.32 | Salt 0.46 | | Salt 0.44 | | | Salt 0.44 | | Defluorinated monocalcium phosphate 0.30 | | Defluorinated monocalcium phosphate 0.30 | |
| Defluorinated monocalcium phosphate 0.30 | Progut. yeast hydrolysate 0.32 | | Defluorinated monocalcium phosphate 0.37 | | | Defluorinated monocalcium phosphate 0.37 | | Coconut oil 0.21 | | Coconut oil 0.21 | |
| Lactose 0.15 | Defluorinated monocalcium phosphate 0.30 | | Progut. yeast hydrolysate 0.30 | | | Progut. yeast hydrolysate 0.30 | | Wheat gluten 0.16 | | Wheat gluten 0.16 | |
| Progres. tall oil fatty acids 0.10 | Lactose 0.15 | | Progres. tall oil fatty acids 0.10 | | | Progres. tall oil fatty acids 0.10 | | Progres. tall oil fatty acids 0.10 | | Progres. tall oil fatty acids 0.10 | |
|  | Progres. tall oil fatty acids 0.10 | | Lactose 0.06 | | | Lactose 0.06 | | Lactose 0.02 | | Lactose 0.02 | |
|  | MFC 1.00 | |  | | | MFC 2.00 | |  | | MFC 2.00 | |
| **Chemical composition** | | | | | | | | | | | |
|  | | Piglet's creep feed | | | Post-weaning feed | | | | | | |
|  | | Pre-weaning (7-22d) | | | 23-35 d | | | | 35-49 d | | |
| Analysis (per kg) | | Control | | MFC | Control | | MFC | | Control | | MFC |
| Moisture (%) | | 12,0 | | 12,0 | 12,0 | | 12,0 | | 12,5 | | 12,5 |
| Crude protein (%) | | 19,0 | | 19,0 | 16,4 | | 16,4 | | 16,6 | | 16,6 |
| Crude fat (%) | | 6,5 | | 6,5 | 5,2 | | 5,2 | | 4,4 | | 4,4 |
| Crude fiber (%) | | 2,7 | | 3,1 | 4,3 | | 4,3 | | 4,0 | | 4,0 |
| Ash (%) | | 5,1 | | 5,1 | 4,8 | | 4,8 | | 4,9 | | 4,9 |
| Methionine (g) | | 5,4 | | 5,4 | 3,9 | | 3,9 | | 4,3 | | 4,3 |
| Lysine (g) | | 14,8 | | 14,8 | 11,3 | | 11,3 | | 12,1 | | 12,1 |
| Calcium (g) | | 7,3 | | 7,3 | 6,6 | | 6,6 | | 6,7 | | 6,7 |
| Phosphorus (g) | | 5,4 | | 5,4 | 5,0 | | 5,0 | | 4,9 | | 4,9 |
| Sodium (g) | | 3,0 | | 3,0 | 3,0 | | 3,0 | | 2,5 | | 2,5 |
| NE (MJ) | | 10,9 | | 10,9 | 10,2 | | 10,2 | | 9,9 | | 9,9 |

**Supplementary Table S5. Partial Illumina TruSeq adapter sequences added to the 5’ ends.**

| Forward | Reverse |
| --- | --- |
| F_1; CCTACGGGNGGCWGCAG | R_1; GACTACHVGGGTATCTAATCC |
| F_2; gtCCTACGGGNGGCWGCAG | R_2; aGACTACHVGGGTATCTAATCC |
| F_3; agagCCTACGGGNGGCWGCAG | R_3; tctGACTACHVGGGTATCTAATCC |
| F_4; tagtgtCCTACGGGNGGCWGCAG | R_4; ctgagtgGACTACHVGGGTATCTAATCC |
